# A Comparative Morphological Study of the Ultrastructure of Antennal Sensilla in *Sclerodermus guani* (Hymenoptera: Bethylidae)

**DOI:** 10.3390/insects16050547

**Published:** 2025-05-21

**Authors:** Youcheng Chen, Chunxia Wang, Xiuju Yu, Bo Wang, Zhudong Liu

**Affiliations:** 1 College of Life Science, Hebei Basic Science Center for Biotic Interactions, Institute of Life Science and Green Development, Hebei University, Baoding 071002, China; chasonchenyc@163.com (Y.C.); cxwang2022@163.com (C.W.); zhuyizhibx@163.com (X.Y.); 2 Xishuangbanna Tropical Botanical Garden, Chinese Academy of Sciences, Menglun, Mengla, Kunming 666303, China

**Keywords:** *Sclerodermus guani*, antennae, olfactory sensilla, morphology, scanning electron microscope

## Abstract

The morphology, number, and distribution of antennal sensilla differ between males and females, reflecting adaptations to sex-specific ecological roles and life histories. In this study, scanning electron microscopy was employed to examine the antennal structure and sensilla types of adult males and females of *Sclerodermus guani* Xiao et Wu 1983 (Hymenoptera: Bethylidae), with a focus on identifying morphological differences between the sexes. The results revealed that the antennae of both sexes are geniculate; however, female antennae are shorter and broader than those of males. Each antenna comprises 13 segments, including a scape (1 segment), a pedicel (1 segment), and a flagellum (11 segments). Eight distinct types of sensilla were identified on the antennae of both males and females, with notable sex-specific differences in sensilla types and subtypes. Trichoid sensilla subtype III was found exclusively in males, whereas long basiconic sensilla and basiconic sensilla subtype II were unique to females. More than 70% of the antennal sensilla in both sexes were olfactory in nature, highlighting their predominant role in chemical detection. The observed sexual dimorphism in the morphology and distribution of olfactory sensilla suggests functional specialization, potentially linked to host localization in females and mate location in males.

## Simple Summary

Sexual dimorphism in life history traits is common among insects and often manifests in distinct sensory adaptations between males and females. In particular, such differences can significantly influence the function and morphology of the antennae, the primary sensory organs involved in environmental perception. In this study, we investigated sexual dimorphism in the antennal structures of *Sclerodermus guani*, a parasitoid wasp widely utilized in biological control programs. Using scanning electron microscopy, we conducted a comparative analysis of antennal morphology and sensilla types between males and females. Our results reveal marked differences in both the structure and distribution of sensilla, indicative of functional specialization: female antennae appear adapted for host detection, while male antennae are likely specialized for mate location. These findings highlight the relationship between antennal architecture and the divergent ecological roles of each sex, contributing to a broader understanding of the evolutionary pressures shaping sex-specific sensory adaptations in insects.

## 1. Introduction

Males and females often occupy distinct microniches and experience divergent life histories, leading to pronounced sexual dimorphism in traits such as body size, coloration, morphology, and physiology [1]. In general, males are primarily challenged with locating food and potential mates, whereas females must navigate a broader array of behavioral demands, including foraging, mate selection, host recognition, and identifying suitable sites for oviposition [2,3]. Insects rely heavily on their antennae as the principal sensory organs for perceiving external stimuli and mediating behavioral responses. Antennae fulfill multiple sensory roles, including olfaction, gustation, mechanoreception, and the detection of thermal and humidity cues [4,5]. The sensory functions are facilitated by specialized structures known as sensilla, which are distributed on the antennal surface [6,7]. Sensilla can be functionally classified into three major categories: chemosensory sensilla (CS), mechanosensory sensilla (MS), and thermo-hydro sensory sensilla (T-HS) [8]. Due to the differing ecological roles and behavioral requirements of each sex, significant variations exist in the types, distribution, and abundance of sensilla between males and females [4,9,10]. Such differences are indicative of sex-specific evolutionary pressures and adaptive strategies shaping the antennae as signal perception and processing [11].

In parasitoid wasps, visual perception is generally limited, making chemical sensing a crucial component of their ecological interactions and life history strategies [12]. These insects can detect a wide range of chemical compounds, enabling them to perceive cues from both conspecifics and hosts within complex environments [13]. Chemical signals employed by parasitoids for host location have been broadly categorized into three main types: plant volatiles, host feces or glandular secretions, and sex pheromones [14]. Notably, olfactory preferences and receptor expression profiles differ significantly between sexes. Female parasitoid wasps typically exhibit heightened sensitivity to host plant volatiles and host-derived secretions, which assist in identifying suitable oviposition sites [15,16,17], whereas males are more sensitive to female-emitted sex pheromones [18,19]. These functional distinctions in host localization and mate recognition are reflected in marked sexual dimorphism in antennal morphology and sensilla composition. Female antennae often display greater morphological complexity and a more diverse array of sensilla types, aligning with their need for a broader spectrum of environmental cues. In contrast, male antennae in certain species are characterized by specialized structures such as tyloids—raised fields that are densely covered with specialized trichoid sensilla containing specific pores, presumably adapted for pheromone detection [20]. Similar patterns of sexual dimorphism have been observed in other insect taxa, including beetles and true bugs, where males possess longer antennae and a higher density of sensilla, some of which are structurally distinct and likely specialized for detecting female sex pheromones [21,22,23]. Interestingly, antennal adaptation in males can also manifest as reduction or degeneration, as observed in fig Wasps, where antennal morphology is simplified due to restricted functional requirements [24]. In many parasitoid species, mating occurs in close proximity to the host, and males may primarily rely on contact pheromones or short-range volatiles for mate localization. Accordingly, male antennae are often adapted for short-range signal detecting, potentially through elongation or the presence of specialized sensilla tuned to sex pheromones [25,26].

*Sclerodermus guani* Xiao et Wu 1983 (Hymenoptera: Bethylidae) is an obligate ectoparasitoid wasp known to parasitize more than 50 host species, primarily targeting the larvae and pupae of various wood-boring insects [27]. This species exhibits typical sexual dimorphism in adults, with distinct differences in morphology and behavior between males and females [28,29]. Due to its parasitic efficacy, *S. guani* has been widely adopted as a biological control agent in integrated pest management programs aimed at controlling wood-boring pests [30]. *S. guani* exhibits a haplodiploid reproductive system, in which fertilized eggs develop into diploid females, while unfertilized eggs develop into haploid males. Adult females are generally larger, wingless, and morphologically adapted for host foraging and parasitism, whereas males are smaller, winged, and primarily focused on mating. Male cocoons typically emerge 1–2 days earlier than those of females, and males actively penetrate female cocoons to copulate immediately upon emergence (personal observation). The progeny are predominantly female even when the mother has mated, suggesting a female-biased sex ratio [31,32]. Female *S. guani* exhibit highly developed host-location capabilities, utilizing their antennae and mandibles to meticulously inspect potential hosts before paralyzing them with their ovipositor stinging [33,34,35,36]. In contrast, males do not participate in host-seeking and die shortly after mating. The pronounced differences in life history strategies and behavioral roles between the sexes are likely reflected in corresponding variation in antennal morphology and sensilla composition, indicative of sex-specific sensory specialization.

Several studies have investigated the antennal morphology of various *Sclerodermus* species using scanning electron microscopy (SEM), including *Sclerodermus sichuanensis* Xiao 1995 (Hymenoptera: Bethylidae) [37], *Sclerodermus alternatusi* Yang 2024 (Hymenoptera: Bethylidae) [38], *Sclerodermus pupariae* Yang et Yao 2012 (Hymenoptera: Bethylidae) [39], and *Sclerodermus cereicollis* Kieffer 1904 (Hymenoptera: Bethylidae) [40]. These studies reveal that, while antennal structures are generally conserved across closely related species, notable sexual dimorphism exists in the types and distribution patterns of sensilla. However, a lack of standardized terminology has led to inconsistencies in the classification and naming of sensilla types across different investigations. Although previous research has addressed the antennae and sensilla features of *S. guani* [28,41], ambiguities remain regarding the precise description, classification, and sexual differentiation of sensilla. Further clarification is therefore needed to enhance our understanding of the sensory adaptations underlying the distinct behavioral roles of males and females in this species.

To enhance our understanding of antennal structure and sensilla distribution in *S. guani* and to provide insights into its chemical ecology, sex recognition, host-searching behavior, and social interactions, a re-examination of antennal ultrastructure is necessary. In this study, scanning electron microscopy (SEM) was employed to examine the antennae of adult females and males, with detailed descriptions of sensilla morphology and statistical comparisons of their types, numbers, and distribution across antennal segments. By comparing these findings with existing data from related *Sclerodermus* species, this research aims to refine the classification of sensilla types and propose functional hypotheses. The results will serve as a foundation for future electrophysiological and behavioral studies, particularly those investigating chemical communication and olfactory mechanisms in this parasitoid wasp.

## 2. Material and Method

### 2.1. Insects

The base stock of *S*. *guani* used in this study was originally obtained from Xishan Forest Farm (Beijing, China), where it was maintained on larvae of *Saperda populnea* Linnaeus 1758 (Coleoptera: Cerambycidae), a commonly used alternative host for mass-rearing purposes. In the laboratory, the parasitoid was subsequently reared exclusively on larvae of *Monochamus alternatus* Hope 1842 (Coleoptera: Cerambycidae) (Ma) for 100 consecutive generations prior to the commencement of this study. *Sclerodermus guani* wasps were reared individually in glass vials (7 cm in height × 1 cm in diameter), each provided with a single host larva for reproduction. Rearing was conducted under controlled environmental conditions in a climate chamber set at 25 ± 0.5 °C, 70% relative humidity, and a 14:10 h light/dark photoperiod, continuing until adult emergence. Newly emerged male and female wasps were preserved in 75% ethanol and stored at 4 °C for subsequent experimental analyses.

### 2.2. Scanning Electron Microscopy (SEM) Photography

Sample preparation and SEM imaging followed the methods described by Yang [42]. Adult *S. guani* specimens preserved in 75% ethanol were sexed, and individuals with intact antennae were selected under a stereomicroscope. Specimens were washed three times with 10% physiological saline and further cleaned in Phosphate Buffered Saline (PBS, Macklin) buffer (pH 7.2) using an ultrasonic cleaner (SK1200H, Shanghai Hank Scientific Instrument Co., Ltd., Shanghai, China) for 60 s. The cleaned samples were then fixed in 2.5% glutaraldehyde (J&K) at 4 °C for 4 h. Post-fixation, samples were rinsed three times with PBS buffer (pH 7.2) and dehydrated through an ethanol series (50%, 75%, 80%, 90%, and 95%), each for 10 min, followed by immersion in 100% ethanol for 20 min. Samples were then transitioned into isoamyl acetate (AMETHYST CHEMICALS) for 15 min and dried using a CO_2_ critical point dryer (K850, Quorum Tech. Ltd., London, UK). Antennae were carefully dissected under a stereomicroscope (XTL-165-VT, Jiangxi Phoenix Optical Technology Co., Ltd., Shangrao, China) and mounted on SEM stubs using double-sided conductive adhesive at dorsal and ventral orientations. The sample was gold-coated using an ion sputter coater (Q150R S, Quorum Tech. Ltd., London, UK) in two 8 min cycles at rotation angles of 90°, 180°, 270°, and 360° to ensure uniform coverage. SEM imaging was performed using an EVO LS10 scanning electron microscope (Carl Zeiss Microscopy Ltd., Cambridge, UK) at a voltage of 10 kV. To account for individual variations, antennae from 10 males and 10 females were imaged.

### 2.3. Sensilla Classification

The nomenclature and classification of sensilla in *S. guani* in this study are primarily based on their external morphology and spatial distribution, following established criteria from previous literature [8,11,43,44]. Additionally, reference was made to earlier descriptions of *S. guani* [28] and other closely related *Sclerodermus* species to ensure consistency and comparability. A comprehensive list of abbreviations for antennal structure and sensilla used in this study is provided in Table 1. Functional inferences for the identified sensilla are based on two key morphological characteristics: (1) the presence or absence of surface pores and (2) the structure of the basal socket, particularly whether it permits flexible movement of the sensillum [45].

### 2.4. Data Analysis

Image enhancement was performed using Adobe Photoshop CC 2023 (v24.2, Adobe Systems Incorporated, San Jose, CA, USA), while morphological measurements of the antennae and sensilla were conducted using Image J (v1.54p, https://wsr.imagej.net/notes.html, accessed on 17 February 2025, National Institutes of Health, Bethesda, MD, USA) [46]. Length and width measurements of the antennae and sensilla were statistically compared using independent sample *t*-tests. For sensilla too small to measure directly, the dimensions of their basal cavities were measured. As the distribution of sensilla counts did not conform to normality, the non-parametric Mann–Whitney U test was employed. All statistical analyses were performed using R 4.3.3 (R Core Team, Vienna, Austria, 2024).

## 3. Results

### 3.1. Comparison of the Antennae Between Female and Male *Sclerodermus guani*

**Figure 1:**
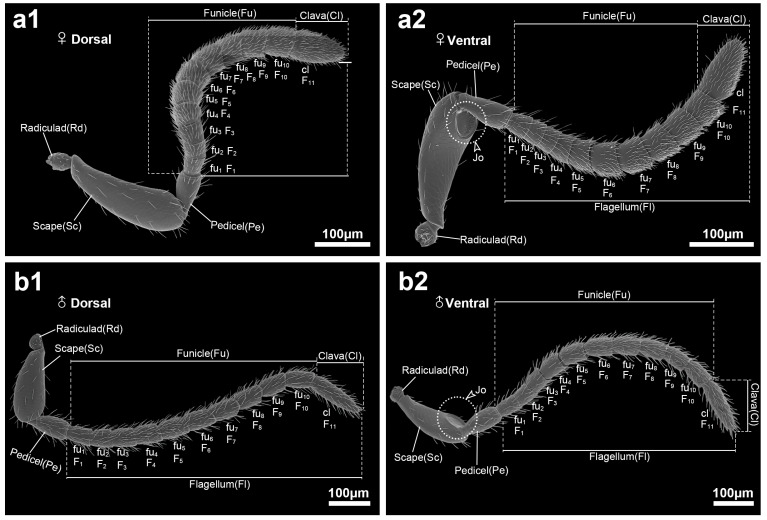
Antennal morphology of female and male *Sclerodermus guani. S*. *guani* female dorsal view (**a1**) and ventral view (**a2**); *S*. *guani* male dorsal view (**b1**) and ventral view (**b2**). Rd, radicula; Sc, scape; Pe, pedicel; Fu, funicle; Cl, clava; Fl, flagellum; F_1_ to F_11_, flagellomere segment 1 to flagellomere segment 11; Jo, wide membranous joint between the scape and the pedicel; scale bar = 100 μm. Abbreviations follow Table 1.

The antennae of both male and female *S. guani* adults are geniculate in form and composed of three primary segments: the scape (Sc), pedicel (Pe), and flagellum (Fl) (Figure 1). The scape is an elongated, cylindrical segment that is slightly curved, tapering at the base and gradually expanding toward the apex. A distinct depression is visible on the ventral side at the articulation between the scape and the connection to the pedicel (see Figure 1 (a2,b2), dotted circle). The radicula (Rd), a short segment, anchors the scape to the head capsule, with its basal end seated in a socket and the apical end connecting to the scape base. The pedicel is also cylindrical, with a narrower basal and a broader distal region. The flagellum, the longest antennal segment, shows the most pronounced sexual dimorphism and constitutes approximately 3/4 of the total antennal length. It comprises 11 flagellomeres and is subdivided into two regions: the funicle (Fu) and the clava (Cl). Each flagellomere is interlocked with the adjacent segment in a sleeve-like configuration. In both sexes, the terminal (11th) flagellomere is expanded into a distinctive, hammer-shaped clava (Figure 1).

**Figure 2:**
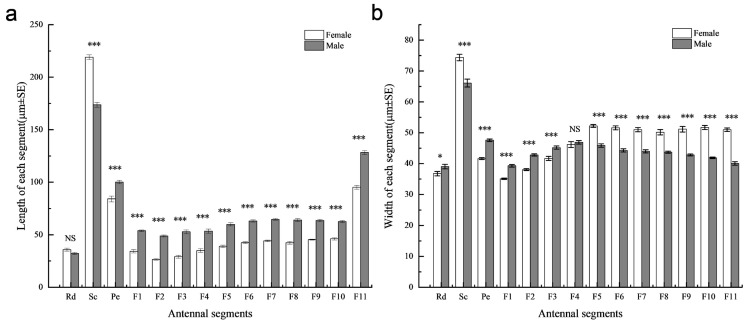
Comparison of the antennal segment length and width between female and male *Sclerodermus guani*. (**a**) The length of each antennal segment; (**b**) the width of each antennal segment. The comparison was conducted using an independent samples *t*-test; NS, *p* > 0.05; *, *p* < 0.05; ***, *p* < 0.001. Columns and bars represented mean ± standard error (*n* = 10). Abbreviations follow Table 1.

The total antennal length of adult *S. guani* females and males was 816.98 ± 7.48 μm and 1033.25 ± 3.96 μm, respectively, indicating a significant difference between the sexes (*t* = −25.55, *d.f.* = 18, *p <* 0.001). No significant difference was observed in the length of the radicula between sexes (*t* = 1.99, *d.f.* = 18, *p* = 0.0617). Interestingly, females exhibited significantly longer scapes than males (*t* = 14.08, *d.f.* = 18, *p <* 0.01), while all other antennal segments were significantly longer in males (Figure 2a). Regarding antennal width, no significant difference was found in the 4th flagellomere between males and females (*t* = −0.68, *d.f.* = 18, *p* = 0.505). However, males exhibited significantly greater width in the radicula (*t* = −2.24, *d.f.* = 18, *p <* 0.05), pedicel (*t* = −12.08, *d.f.* = 18, *p <* 0.001), first flagellomere (*t* = −9.33, *d.f.* = 18, *p <* 0.001), second flagellomere (*t* = −9.94, *d.f.* = 18, *p <* 0.001), and third flagellomere (*t* = −4.13, *d.f.* = 18, *p <* 0.001). Conversely, the remaining flagellomere is significantly wider in females (*p <* 0.001) (Figure 2b; Table S1).

### 3.2. Comparison of the Number and Types of Sensilla Between Female and Male *Sclerodermus guani*

**Figure 3:**
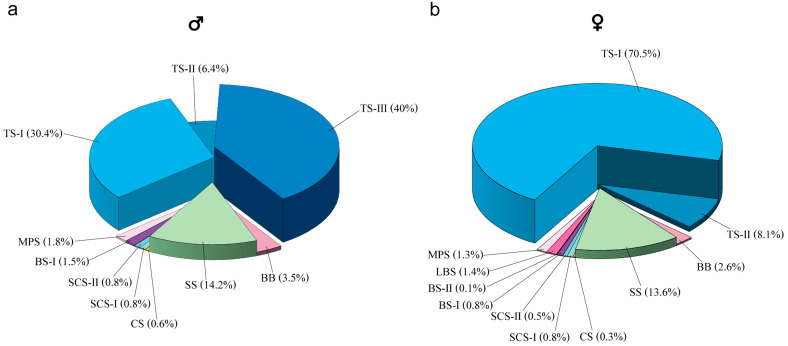
The percentage of each sensilla type in the male (**a**) and female (**b**) *Sclerodermus guani.* Number of sensilla: male, 952.17 ± 4.78 (mean ± standard errors); female, 1276 ± 9.91 (mean ± standard errors). Abbreviations follow Table 1.

A total of eight distinct types of antennal sensilla, comprising 13 categories including subtypes, were identified on the antennae of *S. guani*. Both sexes exhibited relatively fewer sensilla on the scape (Sc) and pedicel (Pe), with the majority of sensilla concentrated on the flagellum (Fl). Each sensillum type exhibited characteristic morphological features and a specific distribution pattern across the antennal segments (Table 2). Detailed morphological descriptions for each sensillum type in males and females are shown in Table 3. The relative proportions of each sensillum type differed notably between male and female *S. guani*, as illustrated in Figure 3 (panels a and b, respectively).

**Figure 4:**
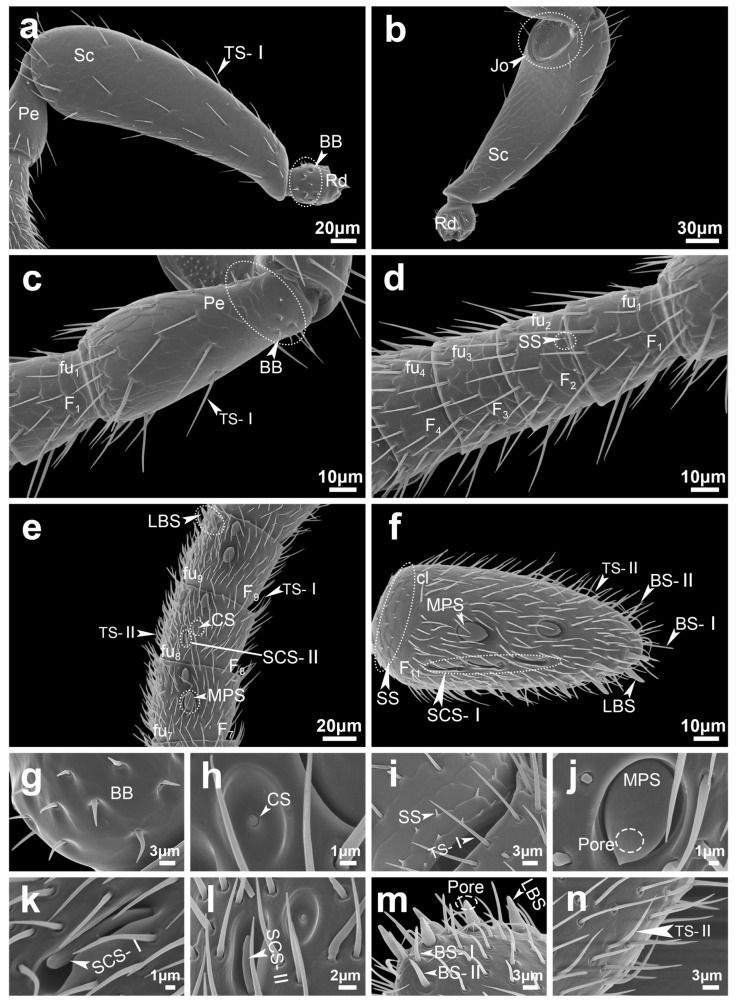
Antennal segment morphology of female *Sclerodermus guani*. Antennal segments include the radicula, scape, pedicel, and flagellum (comprising the funicle and clava), along with the distribution of various types of attached sensilla. (**a**) dorsal morphology of radicula and scape; (**b**) view morphology of radicula and scape; (**c**) pedicel morphology; (**d**,**e**) funicle morphology; (**f**) clava morphology; (**g**) BB morphology; (**h**) CS morphology; (**i**) SS, TS-I morphology; (**j**) MPS morphology; (**k**) SCS-I morphology; (**l**) SCS-II, CS morphology; (**m**) LBS, BS-I and BS-II morphology; (**n**) TS-II morphology. The white line in the lower right corner indicates the scale. Abbreviations follow Table 1.

**Figure 5:**
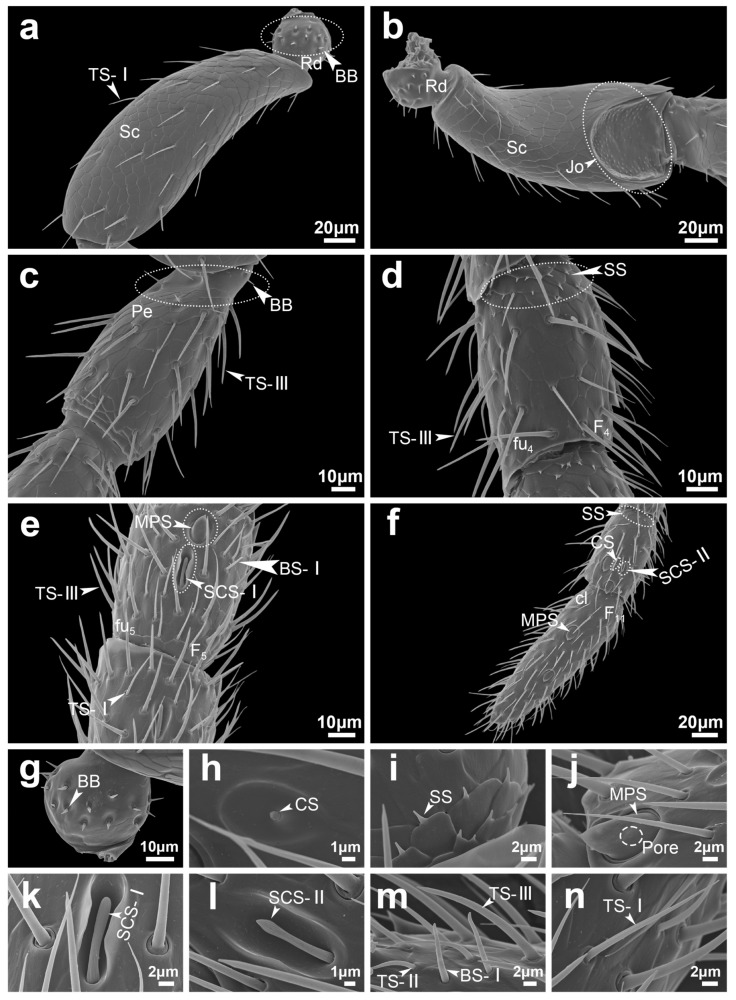
Antennal segment morphology of male *Sclerodermus guani*. Antennal segments include the radicula, scape, pedicel and flagellum (funicle and clava), as well as the distribution of various types of sensilla attached to them. (**a**) dorsal morphology of radicula and scape; (**b**) view morphology of radicula and scape; (**c**) pedicel morphology; (**d**,**e**) funicle morphology; (**f**) clava morphology; (**g**) BB morphology; (**h**) CS morphology; (**i**) SS morphology; (**j**) MPS morphology; (**k**) SCS-I morphology; (**l**) SCS-II morphology; (**m**) TS-II, TS-III, BS-I morphology; (**n**) TS-I morphology. The white line in the lower right corner indicates the scale. Abbreviations follow Table 1.

Böhm bristles (BB) were consistently observed at the articulation between the radicula, scape, and pedicel in both male and female *S. guani* (Figure 4a,c and Figure 5a,c; Table 2). The BBs are short, stiff, spine-like structures that stand nearly perpendicular to the antennal surface. They possess a smooth, non-porous exterior and are anchored within distinct epidermal depressions that feature open basal pits (Figure 4g and Figure 5g). In males, BB measured 5.45 ± 0.48 μm in length and 0.97 ± 0.04 μm in width, while in females, they measured 4.5 ± 0.28 μm in length and 0.94 ± 0.06 μm in width. Statistical analysis revealed no significant differences between the sexes in either length (*t =* 1.71, *d.f.* = 22, *p =* 0.101) or width (*t =* 0.48, *d.f.* = 22, *p =* 0.635) (Table 3). Similarly, the number of BBs did not differ significantly between males and females (U = 18, *p =* 0.699) (Table 2).

**Figure 6:**
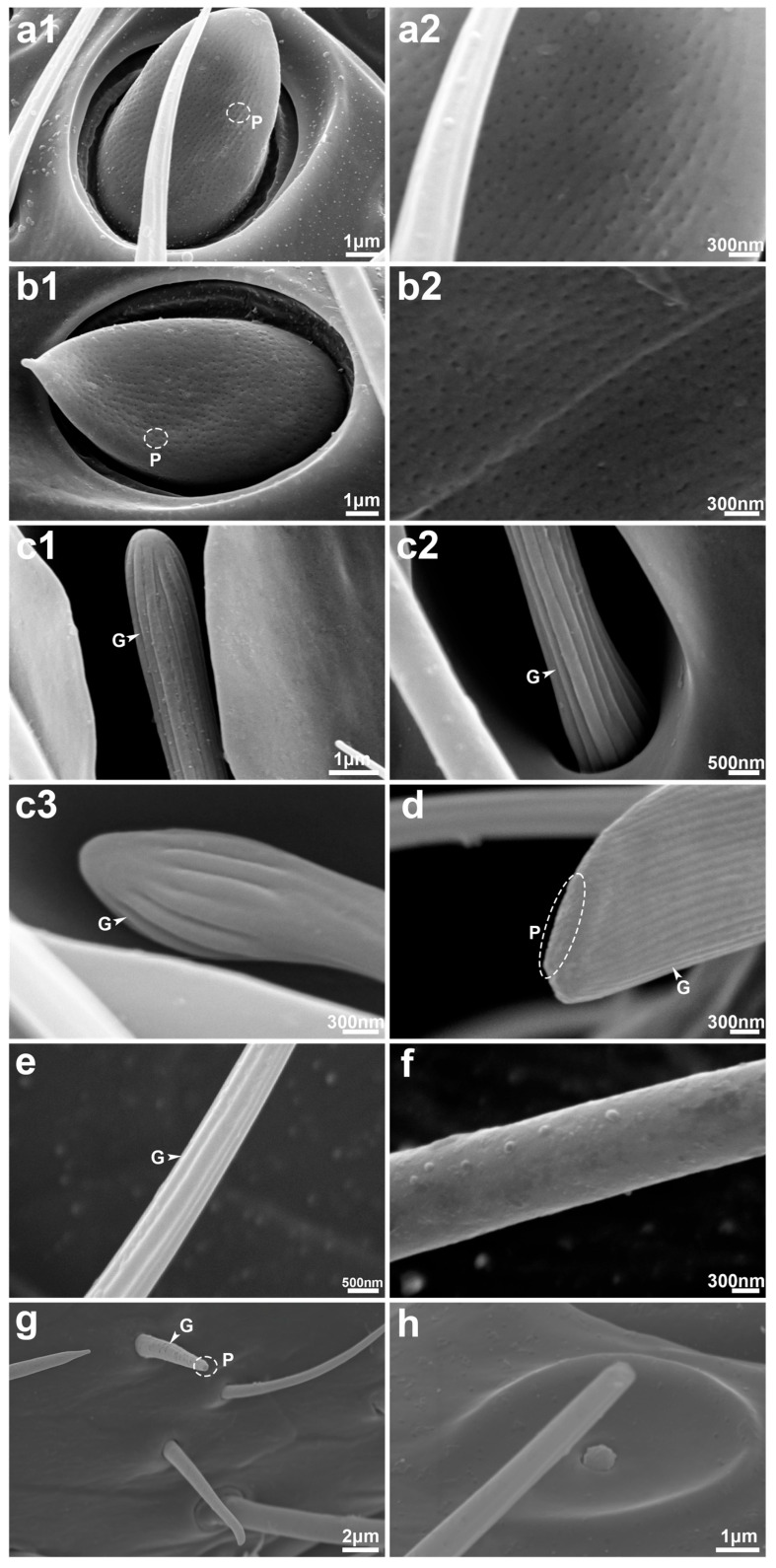
Antennal sensilla surface detail of *Sclerodermus guani.* The multiporous plate sensilla of female (**a1**) and male (**b1**) *Sclerodermus guani;* the surface micropore of multiporous plate sensilla in female (**a2**) and male (**b2**) *Sclerodermus guani.* (**c1**) Styloconic sensilla type I distal portion; (**c2**) Styloconic sensilla type I base; (**c3**) Styloconic sensilla type II distal portion; (**d**) Long basiconica sensilla end portion; (**e**) Trichodea sensilla type I; (**f**) Trichodea sensilla type II; (**g**) Basiconica sensilla type I; (**h**) Coeloconica sensilla. P: pore; G: groove. The white line in the lower right corner indicates the scale.

Trichodea sensilla (TS) are the most abundant sensilla type in both male and female *S. guani*, occurring on all antennal segments except the radicula (Figure 1). Based on morphological characteristics, TS were further classified into three subtypes: TS-I, TS-II, and TS-III. TS-I is broadly distributed across all antennal segments excluding the radicula (Table 2). These sensilla are slender and hair-like, tapering gradually from base to tip. They are anchored in epidermal depressions with open basal pits and display a smooth, non-porous surface marked by longitudinal grooves extending from base to apex (Figure 4i, Figure 5n and Figure 6e). TS-II is restricted to the funicular segments of both sexes (Table 2) and morphologically similar to TS-I but shorter. The basal portion of TS-II is slightly elevated and more constricted than that of TS-I and lacks a closed basal pit. These sensilla lie nearly parallel to the antennal surface and exhibit a slight bend approximately one-third of the way from the base. Like TS-I, they possess a smooth surface devoid of pores and bear longitudinal grooves (Figure 4n and Figure 5m). TS-III is exclusively observed on the pedicel and flagellum of the male antenna (Figure 5c–e; Table 2). It is distinctly longer and thicker than the other subtypes and is rooted in a distinctly raised and fully closed basal pit. A notable ring-like partition separates the base of the sensillum from the antennal surface. The sensilla surface is smooth, lacking both pores and longitudinal grooves (Figure 5m and Figure 6f).

The TS-I sensilla located on the scape and pedicel of both male and female *S. guani* antennae are notably longer than those situated on the flagellum. In males, the TS-I on the scape and pedicel measures 22.35 ± 0.68 μm in length with a base width of 1.18 ± 0.04 μm, while in females, these sensilla measure 23.49 ± 0.71 μm in length and 1.22 ± 0.05 μm in base width. Statistical analysis reveals no significant difference in either length (*t =* −1.16, *d.f.* = 38, *p =* 0.255) or width (*t =* −0.54, *d.f.* = 38, *p =* 0.593) between the two sexes for TS-I on the scape and pedicel (Table 3). In contrast, TS-I sensilla on the flagellum exhibit significant sexual dimorphism. In males, TS-I measures 16.51 ± 0.24 μm in length and 0.97 ± 0.03 μm in base width, whereas in females, these values are 13.22 ± 0.35 μm and 0.82 ± 0.03 μm, respectively. Both the length (*t =* 7.64, *d.f.* = 38, *p <* 0.01) and base width (*t =* 3.66, *d.f.* = 38, *p <* 0.01) of TS-I on the flagellum are significantly greater in males than in females (Table 3). However, the total number of TS-I is significantly higher in female antennae compared to males (U = 0, *p =* 0.002) (Table 2).

The TS-II sensilla on male *S. guani* antennae measure 16.07 ± 0.45 μm in length and 0.87 ± 0.01 μm in base width. In females, TS-II sensilla measure 11.88 ± 0.19 μm in length and 0.86 ± 0.02 μm in base width. Statistical analysis indicates that TS-II sensilla are significantly longer in males than in females (*t =* 8.58, *d.f.* = 26, *p <* 0.01), while no significant difference in base width is observed between the sexes (*t =* 0.49, *d.f.* = 26, *p =* 0.631) (Table 3). However, the number of TS-II sensilla is significantly greater on female antennae compared to males (*U* = 0, *p =* 0.002) (Table 2). TS-III, observed exclusively on male antennae, measures 24.34 ± 0.47 μm in length with a base width of 2.06 ± 0.05 μm (Table 3). Among all sensilla types, trichodea sensilla represent the largest proportion on the antennae, accounting for 78.7% in females and 76.8% in males (Figure 3).

Multiporous plate sensilla (MPS) are located on the ventral surface of the flagellomeres of both male and female *S. guani* antennae (Figure 4e,f and Figure 5e,f; Table 2). These sensilla are situated within distinct, circular epidermal depressions. Morphologically, MPS are oval-shaped, with their basal ends embedded within the groove and their apices protruding outward. The apex tapers and is covered with numerous micropores. MPS are typically interspersed among TS, CS, and SCS (Figure 4j, Figure 5j and Figure 6(a1,a2,b1,b2)). The apical morphology differs between sexes: in males, the MPS apex is pointed and cone-like, while in females it is blunt and rounded. Sex-specific differences in MPS distribution were observed: in males, MPS are distributed on flagellomeres F3 to F11, whereas in females they are present on F4 to F11 (Table 2). The length of MPS in males is significantly greater (11.25 ± 0.24 μm) than in females (10.19 ± 0.27 μm) (*t =* 2.94, *d.f.* = 26, *p <* 0.01), although no significant difference in width is observed between the sexes (6.29 ± 0.14 μm in males vs. 5.89 ± 0.21 μm in females; *t =* 1.64, *d.f.* = 26, *p =* 0.113) (Table 3). The number of MPS does not differ significantly between males and females (U = 18, *p =* 1.0) (Table 2).

Coeloconica sensilla (CS) are present on the flagellomeres of both male and female *S. guani* antennae, with a single sensillum observed per segment (Figure 4e and Figure 5f; Table 2). Morphologically, CS appear as short, nipple-like projections centrally positioned with broad, circular epidermal depressions. Their surfaces are marked by irregular grooves (Figure 4h,l, Figure 5h and Figure 6h). Notable sexual dimorphism exists in the distribution of CS: in males, they are located on flagellomeres F5 to F10, while in females, they are restricted to flagellomeres F7 to F10 (Table 2). Measurements show that the cavity length of CS in males (7.27 ± 0.19 μm) is significantly greater than in females (6.64 ± 0.19 μm) (*t =* 2.32, *d.f.* = 10, *p <* 0.05). However, the cavity width does not significantly differ between sexes (4.41 ± 0.22 μm in males vs. 3.97 ± 0.16 μm in females; *t =* 1.61, *d.f.* = 10, *p =* 0.139) (Table 3). The number of CS is also significantly higher in males than in females (U = 36, *p =* 0.002) (Table 2).

Squamiform sensilla (SS) are located at the junctions between flagellomeres in both male and female *S. guani* antennae and are broadly distributed from the second flagellomere to the clava (Figure 4d and Figure 5d; Table 2). Morphologically, SS are scale-shaped structures that emerged from the patterned epidermal surface and adhere to the antenna. Each sensillum features a relatively broad base that tapers to a short conical shape (Figure 4i and Figure 5i). In males, SS measures 1.69 ± 0.25 μm in length and 0.75 ± 0.08 μm in width, whereas in females, they are shorter and narrower (0.91 ± 0.16 μm in length and 0.54 ± 0.06 μm in base width). These differences are statistically significant for both length (*t =* 4.29, *d.f.* = 28, *p <* 0.01) and width (*t =* 3.97, *d.f.* = 28, *p <* 0.01) (Table 3). Interestingly, despite their smaller dimensions, female antennae possess significantly more SS than those of males (U = 0, *p =* 0.002) (Table 2). In terms of overall abundance, SS represent the second most common sensilla types, comprising 13.6% and 14.2% of total antennal sensilla in females and males, respectively. Combined with trichodea sensilla (TS), TS and SS account for over 80% of all sensilla.

Basiconica sensilla (BS) are morphologically classified into two subtypes: BS subtype I (BS-I) and BS subtype II (BS-II). BS-I occurs in both male and female *S. guani* antennae but exhibits sexually dimorphic distribution. In males, BS-I is distributed on the ventral surface of flagellomeres F4 to F10, whereas in females, it is restricted to the ventral side of the clava (Figure 4f and Figure 5e; Table 2). Morphologically, BS-I is short and conical, with a broad base that tapers gradually toward the tip. The sensillum is anchored in a distinctly raised, closed pit and extends perpendicularly from the antenna surface. Its surface is marked by multiple longitudinal grooves (Figure 4m, Figure 5m and Figure 6g). In males, BS-I measures 9.93 ± 0.13 μm in length and 1.03 ± 0.04 μm in base width, whereas in females, it measures 8.38 ± 0.39 μm in length and 1.05 ± 0.05 μm in width. The difference in length between sexes is statistically significant (*t =* 3.71, *d.f.* = 22, *p <* 0.01), while the width shows no significant difference (*t =* −0.33, *d.f.* = 22, *p =* 0.743) (Table 3). Males also possess a significantly higher number of BS-I than females (U = 36, *p =* 0.002) (Table 2). BS-II, by contrast, is exclusively observed at the apex of the clava in female antennae, with only a single instance recorded. This subtype is more robust than BS-I, with a broader base and a more pronounced taper toward the tip. Unlike BS-I, the surface of BS-II lacks longitudinal grooves and appears smooth. BS-II measures 11.08 ± 0.23 μm in length and 1.70 ± 0.02 μm in width (Table 3).

Long basiconica sensilla (LBS) are exclusively observed on the ventral side of the distal part of flagellomere F6 to the clava in female antennae (Figure 4e,f; Table 2). Morphologically similar to BS, LBS are generally more robust, featuring a broader base that gradually tapers toward the apex. Each LBS is anchored within a prominently elevated, closed pit, marked by a distinct ring-like partition at the base separating it from the antennal surface. The apex of the sensillum is blunt with a relatively flat, beveled surface. The shaft bears multiple longitudinal grooves, and the apical region contains micropores (Figure 4m and Figure 6d). LBS measures 8.15 ± 0.27 μm in length and 2.45 ± 0.05 μm in base width (Table 3).

Styloconic sensilla (SCS) are present on the antennae of both males and females and can be classified into two subtypes: subtype I (SCS-I) and subtype II (SCS-II). The distribution patterns of these subtypes differ between sexes. For males, SCS-I and SCS-II are distributed on flagellomeres F3 to F10. In contrast, in females, SCS-I occurs on flagellomeres F4 to F11, while SCS-II is restricted to F4 to F10 (Figure 4e,f and Figure 5e,f; Table 2).

SCS-I exhibits finger-like morphology, with the base and tip having approximately equal width and a bluntly rounded apex. The sensillum is anchored in a recessed pit, and its surface bears several longitudinal grooves that extend from the base toward the tip, though they do not reach the apex. Notably, SCS-I is surrounded by two thick, disk-like protrusions of the antenna surface, giving the structure a characteristic dumbbell-like appearance (Figure 4k, Figure 5k and Figure 6(c1,c2)).

SCS-II exhibits a mushroom-like morphology, consisting of a slender stalk and a swollen, nail-like tip. The base is embedded in a recessed pit and is relatively narrow, expanding toward the more pointed apex, distinguishing it from the blunt-ended SCS-I. The stalk surface is predominantly smooth, with longitudinal grooves restricted to the tip region. Similarly to SCS-I, SCS-II is surrounded by thick, dumbbell-shaped cuticular protrusions; however, the surrounding structure is more open in comparison (Figure 4l, Figure 5l and Figure 6(c3)).

In male antennae, the length of SCS-I is 12.32 ± 0.18 μm, with a base width of 1.74 ± 0.02 μm, while SCS-II measures 6.96 ± 0.19 μm in length and 0.93 ± 0.02 μm in base width. In female antennae, SCS-I is 12.77 ± 0.16 μm long with a base width of 1.29 ± 0.11 μm, and SCS-II is 7.05 ± 0.13 μm long with a base width of 0.92 ± 0.02 μm. The length of SCS-I does not differ significantly between sexes (*t =* −1.87, *d.f.* = 14, *p =* 0.083), but the base width of SCS-I is significantly wider in male antennae compared to females (*t =* 4.26, *d.f.* = 14, *p <* 0.01). The length (*t =* −0.39, *d.f.* = 18, *p =* 0.697) and base width (*t =* 0.76, *d.f.* = 18, *p =* 0.452) (Table 3).

The number of SCS-I is significantly greater in female antennae than in males (U = 18, *p =* 0.002), whereas the number of SCS-II is significantly greater in male antennae compared to females (U = 18, *p =* 0.002) (Table 2).

Additionally, there is no significant difference between sexes in the proportion of sensilla associated with odor detection and temperature and humidity sensing (TS-I, TS-III, MPS, CS, LBS, BS, SCS) across the entire antenna, with females accounting for 75.7% and males accounting for 75.9%) (*χ*^2^ = 0.26, *d.f.* = 1, *p* = 0.61) (Figure 3).

## 4. Discussion

In this work, we comprehensively characterized the fine morphology of the antennal sensilla of *S*. *guani*, with a comparative analysis between females and males. The overall morphology of antennal sensilla in *S. guani* exhibits notable similarities to that reported in related *Sclerodermus* species in previous studies (Table S2).

Antennae of *S. guani* exhibit no significant sexual dimorphism in overall structure, with both male and female possessing 13 segments, comprising a scape, pedicel, and 11 flagellomeres. Male antennae are generally longer but narrower than those of females. In total, 12 types and subtypes of sensilla, classified into eight categories, were identified in both sexes. Of these, 10 types/subtypes were present in males and 11 in females. TS-III was found exclusively in males, whereas LBS and BS-II were unique to females. Among the shared sensilla types, TS-I, TS-II, and SCS-I were more numerous in females, whereas BS-I, SCS-II, and CS were more abundant in males, indicating sex-specific differences in sensilla distribution.

Previous studies on the antennae of *S. guani* [41] and *Sclerodermus Sichuanensis* [47] classified the antennae as consisting of 14 segments. However, when compared with established insect antennal structures and nomenclature [8,43,48], it appears that these studies mistakenly identified the radicula—typically considered an appendage of the scape—as a distinct segment. Consequently, the original scape and pedicel were reclassified as the pedicel and first flagellomere, respectively, resulting in the 14-segment interpretation. We propose that the antennal segmentation and terminology for *S. guani* should be standardized to align with those used for *Sclerodermus pupariae* [49], *Sclerodermus alternatusi* [50], and *Sclerodermus cereicollis* [51], thereby supporting a 13-segment classification.

By comparing *S. guani* with closely related *Sclerodermus* species, we inferred the potential functions of various antennal sensilla, including roles in mechanoreception, chemoreception, thermos-hydro reception, and CO_2_ detection. The observed sexual dimorphism in antennal length is consistent with findings in related species such as *S*. *pupariae* [49] and *S*. *alternatusi* [50]. Although *Sclerodermus* species exhibit highly similar antennal architectures, notable variations exist in sensilla nomenclature, distribution, and sex-specific differentiation—particularly among chemosensory sensilla. Previous research on the olfactory sensilla of *S. guani* has reported the immunolocalization of two odorant-binding proteins (OBPs) and one chemosensory protein (CSP) [28]. Specifically, OBP1 was expressed in LBS, TS-I, TS-III, and MPS; OBP2 in SCS-II and CSP in MPS, SCS-I, and CS.

### 4.1. Böhm Bristles (BB)

Böhm bristles (BB) were identified on the antennae of *S. guani*. Sensilla with similar morphology and located in comparable antennal positions have also been observed in *S. alternatusi* and *S. pupariae*, where they were identified as Bohm’s Bristles and sensilla chaetica 2, respectively. However, this sensillum type has not been reported in *S. cereicollis* [51]. In some studies, BB has been categorized as a subtype of the spine-like sensilla [43,52], but due to its relatively shorter and simpler structure, many researchers regard it as an independent sensillum type [24,53,54]. Based on morphological comparisons across various Hymenoptera species [24,54,55,56], we confirmed that these sensilla represent a consistent type. Functionally, BB acts as a mechanoreceptor, enabling parasitic wasps to detect optimal host positions and to receive mechanical stimuli during flight, thereby regulating behavior [57,58]. Additionally, studies have shown that ablation of BBs at the antennal base can result in uncoordinated movements and collisions between the antennae and wings [59]. In our study, no differences were observed in the position, distribution, or number of BBs between male and female *S. guani*, suggesting similar mechanosensory capabilities in both sexes.

### 4.2. Trichoid Sensilla (TS)

TS are the most abundant sensilla type on the antennae of *S. guani*, comprising three subtypes (I, II, and III) that exhibit distinct sexual dimorphism. In *S. guani*, the morphological characteristics of TS-I closely resemble those of Sensilla trichodea 1 and Sensilla trichodea 2 in *S. alternatusi*—which differ primarily in length and are considered variations in a single type [50]—as well as the Trichoid sensilla in *S. cereicollis* [51] and Sensilla chaetica 1 in *S. pupariae* [49]. These sensilla are characterized by longitudinal grooves along their surface and a well-defined concave base, with no apparent micropores. Although Sensilla trichodea 2 in *S. pupariae* displays similar grooves and a concave base, micropores were also absent. Transmission electron microscopy (TEM) photos have shown that the cross-section of this sensilla lacks stomata and channels and features a thick, elastic articular membrane (AM) between the tubular body (TB) and the sensillar socket (SS). These characteristics suggest a primary function in mechanoreception rather than chemoreception [51]. However, evidence of OBP1 expression in TS-I [28] suggests a possible role in chemical signal detection as well. This indicates that TS-I may serve both mechanosensory and chemosensory functions, with functional emphasis potentially varying across different *Sclerodermus* species.

Both male and female *S. guani* possess TS-II, whose morphological characteristics closely resemble those of Sensilla trichodea 3 in *S. alternatusi* [50]. These sensilla feature a smooth surface devoid of micropores and exhibit an extremely curved base. Over two-thirds of each sensillum lies parallel and closely attached to the antenna surface, with the base exhibiting slight contraction and low elevation. Given their hair-like, poreless structure, TS-II is generally considered to serve a mechanosensory function [2]. While both sexes exhibit TS-I and TS-II, females have a significantly greater number of these sensilla. It is hypothesized that post-emergence, female *S. guani* spend more time than males actively searching for hosts in complex environments. The greater abundance of TS in females may enhance their ability to detect air currents, substrate vibrations, and other mechanical stimuli critical for host localization and navigation. Notably, the predominance of TS-I in females—comprising 70.5% of total antennal sensilla—likely reflects their specialized behaviors’ requirement for host detection and orientation.

TS-III exhibits morphological characteristics similar to those of Sensilla basiconica 2 in *S. alternatusi* (reported exclusively on male antennae) [50], Sensilla trichodea 3 in *S. pupariae* [49], and Multiporous sensilla chaetica in *S. cereicollis* [51]. This sensillum is longer and slightly curved, with a thicker and more robust structure than typical trichoid sensilla. A key distinguishing feature is its completely closed base socket. Previous studies on Sensilla trichodea 3 and Multiporous sensilla chaetica have reported the presence of surface micropores. Furthermore, these sensilla types, restricted to male antennae, were found to contain distinct dendritic branches (DB), pores, and pore tubules (PT) in cross-sectional analyses, supporting their role in chemosensory functions [28,51]. In the present study, no surface micropores were observed on TS-III in *S. guani*; however, based on morphological comparisons with the existing literature, these structures were classified as the same sensilla type. TS-III is exclusively found on male *S. guani* antennae, distributed across all segments except the scape (including the radicula), and comprises approximately 40% of total sensilla types in males. This distribution pattern suggests a potential role in sex pheromone detection.

Overall, notable sexual dimorphism was observed in the differentiation of TS types in *S. guani*. While TS-I and TS-II were observed in both sexes, males exhibited longer but fewer sensilla compared to females. In contrast, TS-III was exclusively found on male antennae. Despite differences in the types and quantities of individual sensilla, the overall proportion of TS did not differ substantially between sexes. The high prevalence of TS-III in males may be associated with navigation in complex environments, where increased friction and mechanical stimulation to the antennae are likely encountered. These findings support the hypothesis that TS-III represents a male-specific sensillum, potentially functioning in mate localization.

### 4.3. Squamiform Sensilla (SS)

Squamiform sensilla are a relatively uncommon type of sensillum in parasitic wasps. In *S. guani*, *S. sichuanensis*, *S. alternatusi*, and *S. pupariae*, shares exhibit consistent morphological characteristics. The MIC (microtrichia sensilla) described by Zhang [60] and Yang [61] display similarities to the SS observed in this study, particularly in terms of morphology and widespread distribution. These sensilla probably are hypothesized to function in anchoring antennal segments and protecting the antennal surface [62]. Positioned at the junctions between flagellomeres on both male and female antennae, SS may also play a role in detecting mechanical vibrational signals produced during host contact [63]. A statistically significant difference in SS number between males and females was observed, with females exhibiting a greater abundance. Their placement at flagellomere junctions further suggests a potential function in reducing intersegmental friction. This regulatory function may contribute to the enhanced behavioral complexity observed in females. It is hypothesized that the more complex behavioral environments encountered by females result in increased sensory stimulation of the antennae, driving the evolution of a higher number of SS to meet these demands.

### 4.4. Multiporous Plate Sensilla (MPS)

MPS are a common type of olfactory sensillum found on the antennae of Hymenoptera insects [64]. In *S. guani*, the MPS are oval-shaped, consistent with those observed in closely related *Sclerodermus* species. The distinction between blunt and pointed tips on female and male antennae also aligns with findings in *S. cereicollis* [51]. However, the morphology of MPS in *S. guani* differs notably from descriptions in previous studies on other parasitic Hymenoptera [42,54,65,66].

SEM revealed numerous micropores on the surface, while TEM showed pore tubules and sensory neurons with dendritic branches, features indicative of a chemosensory function [51]. Electrophysiological experiments have confirmed that MPS respond to volatile compounds in a concentration-dependent manner [52]. Additionally, research on *Microplitis mediator* Haliday 1834 (Hymenoptera: Braconidae) found high expression of the ion receptor MmIR8a protein in the dendrites of MPS neurons, further supporting their role in olfaction [67].

Electrophysiological studies on three aphid species (Hemiptera) showed that their LP closely resembled the MPS observed in *S. guani* and exhibited a strong response to terpenoid and derivative compounds [68]. In *S. guani*, no significant difference was found in the number of MPS between males and females, suggesting a conserved function across sexes. However, slight discrepancies in their distribution may reflect differing environmental exposures or ecological roles during development.

### 4.5. Long Basiconic Sensilla (LBS)

LBS in *S. guani* exhibit external morphological characteristics with sensilla basiconica 1 in *S. alternatusi*, sensilla basiconica in *S. pupariae*, and long sensilla basiconica in *S. cereicollis* and *S. sichuanensis* [47,49,50,51]. These sensilla are likely homologous and should be classified as the same sensillum type, distinguished by the presence of numerous micropores concentrated at the tip.

TEM observations of LBS (long sensilla basiconica in *S*. *guani*, long sensilla basiconica in *S. cereicollis*) revealed distinct terminal pores in longitudinal sections, as well as a dense concentration of olfactory neurons with dendritic branches within the sensillar lymph cavity in cross-sectional views [28,51]. The outer dendritic segments (ODS) and inner dendritic segments (IDS) are interconnected via ciliary constrictions (CC) [28,51], confirming the chemosensory function of LBS. As this sensillum is found exclusively on female antennae, it is presumed to play a crucial role in host location. Its structure—featuring micropores at the tip and along the shaft—and its positioning on the ventral side of the antenna segments and the distal end of the flagellum suggest that it functions as a contact chemoreceptor. Previous studies have indicated that this type of sensillum is involved in detecting sexual kairomones from host feces and cuticular hydrocarbons (CHCs) from host larvae in *Bethylidae* [15].

### 4.6. Basiconica Sensilla (BS)

In *S. guani*, BS are classified into two subtypes: BS-I and BS-II. The morphology of BS-I resembles that of sensilla trichodea 2 in *S. pupariae* [49] and uniporous grooved sensilla chaetica in *S. cereicollis* [51]. These sensilla are conical, oriented perpendicularly to the antennal surface, and feature longitudinal grooves. In previous studies on *S. guani* [28], this sensillum was identified as sensilla trichodea (ST), which corresponds to the BS-I type described here, with micropores at the tip. Longitudinal section reveals a tubular structure housing a basal neuron, with dendritic branches enclosed in a sheath extending toward the tip. Cross-sections show six neurons [51], indicating a chemosensory function. The distribution of BS-I differs between sexes: in females, it is concentrated on the ventral side of the clava, while in males, it is scattered from F4 to F10. Combined with its perpendicular orientation, this suggests a possible role in tactile or gustatory perception during host contact. BS-II, found exclusively on female antennae, resembles sensilla trichodea 1 in *S. pupariae* and also displays fine micropores on its surface. Although this sensillum was not reported by Li et al. [28], its restricted localization at the tip of the female flagellum and solitary presence suggest it may function in chemoreception, potentially involved in detecting host surface chemicals at close range.

### 4.7. Styloconic Sensilla (SCS)

In *S. guani*, SCS are classified into two subtypes: SCS-I and SCS-II. SCS-I has been described under various names in other studies, including *S. sichuanensis* (styloconic sensillum II), *S. alternatusi* (sensilla styloconica 1), *S. pupariae* (sensilla styloconica 1), and *S. cereicollis* (grooved sensilla ampullacea) [47,50,51]. SCS-II is also present in other *Sclerodermus* species under different names, including *S. sichuanensis* (styloconic sensillum I), *S. alternatusi* (sensilla styloconica 2), *S. pupariae* (sensilla styloconica 2), and *S. cereicollis* (grooved peg sensilla) [47,50,51].

This study diverges from previous research [41] in the classification of styloconica sensilla. Earlier studies did not identify subtypes in *S. guani*, and Li et al. [28] concluded that double-walled multiporous sensilla I (DWPS-I, equivalent to SCS-I) occurred exclusively in females, while double-walled multiporous sensilla II (DWPS-II, equivalent to SCS-II) were present in both sexes. In *S. alternatusi*, SCS-I (as sensilla styloconica 1) and SCS-II (as sensilla styloconica 2) are sex-specific—found exclusively in females and males, respectively. In contrast, both SCS-I and SCS-II are present in both sexes of *S. cereicollis* and *S. pupariae.*

TEM cross-sectional observations of SCS-I (double-walled multiporous sensilla I in *S. guani*) reveal a dendritic branch at the base and, distally, 14 dendritic branchlets enclosed by a double wall within the sensillar lymph cavity [28]. In SCS-I (grooved sensilla ampullacea in *S. cereicollis*), distal cross-sections show pores with microtubes in the cuticular groove, and proximal sections reveal clear dendritic branches within the lymph cavity [51], supporting their chemosensory function. In contrast, SCS-II (double-walled multiporous sensilla II in *S. guani*) shows three neurons in distal cross-section [28], while SCS-II (grooved peg in *S. cereicollis*) contains six neurons [51]. Although both sensilla types possess an inner wall (IW) and an outer wall (OW), no distinct pores were observed in the cuticle connecting the neurons to the external environment, suggesting they do not serve an olfactory function. Some studies propose that these sensilla may instead be involved in thermo-hydro reception [69].

In *S. guani*, SCS-I is more abundant in females and primarily distributed on the flagellum, while SCS-II is more numerous in males. These patterns suggest that SCS-I may be involved in host location, whereas SCS-II likely plays a role in thermo-hydro reception.

### 4.8. The Coeloconica Sensilla (CS)

CS in *S. guani* is a minute sensillum type, morphologically resembling sensilla coeloconica in *S. alternatusi* and *S. pupariae*, the coeloconic peg in *S. cereicollis*, and the campaniform sensillum in *S. sichuanensis*. These structures are characterized by a small, peg-like protrusion situated within a circular depression in the cuticle, suggesting they likely represent the same sensillum type across species. The surface exhibits fine folds and micropores. Cross-sectional observations reveal a curved, elongated chitinous tube beneath the sensillum, with a peg-like structure oriented parallel to the antennal surface. At the distal end, a dendritic sheath (DS) is visible within the sensilla lymph cavity (SL), while the proximal end shows a sheet-like neuron [28,51].

The function of this sensillum remains debated. In *Drosophila melanogaster*, CS exhibited a strong electrophysiological response to acidic amines, and 15 ionotropic receptors (DmIRs) were expressed in these sensilla [70,71], supporting a chemosensory role. Based on differences observed between distal and proximal cross-sections and comparisons with sensillum cell types I and II as defined by Altner and Loftus [69], this sensillum is also believed to be involved in humidity and temperature perception. In this study, CS were more numerous on the male antennae than on the female antennae. Since males eclose earlier and enter the female cocoon to mate—an environment likely to have elevated CO_2_ concentration—it is hypothesized that CS may play a role in sensing temperature, humidity, and CO_2_.

## 5. Conclusions

Our study revealed notable differences between the antennae and sensilla of male and female *S. guani*. Female antennae are shorter but significantly wider than those of males. Analysis of sensilla distribution across antennal segments shows that females possess a greater diversity and higher number of sensilla types—including female-specific types such as LBS and BS-II—likely associated with their foraging behavior. In contrast, the male-specific TS-III sensillum is presumed to be involved in mate-searching behavior. In terms of sensilla composition, over 70% of the sensilla on both male and female antennae are related to chemical perception, indicating that olfaction plays a primary role in host location and pheromone detection.

Notably, while the number and type of sensillum associated with odor, temperature, and humidity detection differ between males and females, the overall proportion of sensilla remains comparable between sexes. This indicates a clear pattern of antennal differentiation, likely reflecting sex-specific odor recognition capabilities. Males emerge earlier than females and exhibit pronounced differences in body size, which may be linked to developmental timing plasticity. This plasticity influences the differentiation of antennae structures and sensilla, ultimately shaping their morphology and function [72]. However, despite identifying a wide variety of sensilla based on external morphology in this polyphagous parasitoid, the precise functions of many sensilla remain to be confirmed through electrophysiological, neurobiological, and gene expression studies.

Naming conventions for insect antennal sensilla also vary widely among researchers. A standardized nomenclature system will require a deeper understanding of sensilla functions to enable clearer classifications. Additionally, systematic studies combining sensilla morphology across taxa with phylogenetic analyses will be essential for resolving current inconsistencies and improving classification frameworks.

## Figures and Tables

**Table 1:** Terminology and abbreviations of antennal segments and sensilla.

Abbreviation	Name
Rd	Radicula
Sc	Scape
Pe	Pedicel
Fu	Funicle
Cl	Clava
Fl	Flagellum
Jo	Wide membranous joint between the scape and the pedicel
fu_1_-fu_10_	1–10 Funicle
F_1_-F_11_	1–11 Flagellomeres
BB	Böhm bristles
TS-I	Trichodea sensilla type I
TS-II	Trichodea sensilla type II
TS-III	Trichodea sensilla type III
MPS	Multiporous plate sensilla
CS	Coeloconica sensilla
SS	Squamiform sensilla
BS-I	Basiconica sensilla type I
BS-II	Basiconica sensilla type II
LBS	Long basiconica sensilla
SCS-I	Styloconic sensilla type I
SCS-II	Styloconic sensilla type II

Note: The abbreviations used here and in the main text are consistent.

**Table 2:** Abundances and distribution of sensilla on the antennae of male and female *Sclerodermus guani*.

Sensilla	Subtype	Genders	Rd	Sc	Pe	Flagellomeres											Total	*p*
						F_1_	F_2_	F_3_	F_4_	F_5_	F_6_	F_7_	F_8_	F_9_	F_10_	F_11_		
BB	-	**♂**	22 ± 0.42	11 ± 0	-	-	-	-	-	-	-	-	-	-	-	-	33 ± 0.42	0.699
		**♀**	22 ± 0.31	11 ± 0	-	-	-	-	-	-	-	-	-	-	-	-	33 ± 0.31	
TS	I	**♂**	-	46 ± 0.56	14 ± 0.23	11 ± 0.56	12 ± 0.48	11 ± 0.48	14 ± 0.56	14 ± 0.7	17 ± 0.49	23 ± 0.58	25 ± 0.31	25 ± 0.67	33 ± 0.92	45 ± 1.07	290 ± 2.23	<0.01
		**♀**		54 ± 1.12	40 ± 1.45	30 ± 0.6	40 ± 1.17	49 ± 1.1	68 ± 1.41	75 ± 1.89	71 ± 1.37	82 ± 1.2	83 ± 1.51	85 ± 1.08	84 ± 2	141 ± 1.94	901 ± 8.5	
	II	**♂**	-	-	-	-	-	-	4 ± 0.21	7 ± 0.33	8 ± 0.21	7 ± 0.34	7 ± 0.26	7 ± 0.42	8 ± 0.22	14 ± 0.42	61 ± 1.05	<0.01
		**♀**							8 ± 0.31	11 ± 0.33	12 ± 0.54	12 ± 0.42	12 ± 0.48	12 ± 0.48	14 ± 0.26	22 ± 0.62	103 ± 1.33	
	III	**♂**	-	-	31 ± 0.72	24 ± 0.8	26 ± 1.2	29 ± 1.18	32 ± 0.61	30 ± 0.9	33 ± 1.15	31 ± 0.76	33 ± 0.77	31 ± 0.68	32 ± 0.89	51 ± 1.15	382 ± 6.04	-
		**♀**			-	-	-	-	-	-	-	-	-	-	-	-	-	
MPS	-	**♂**	-	-	-	-	-	1 ± 0	2 ± 0	2 ± 0	2 ± 0	2 ± 0	2 ± 0	2 ± 0	2 ± 0	2 ± 0	17 ± 0	1
		**♀**						-	1 ± 0	1 ± 0	2 ± 0	2 ± 0	2 ± 0	2 ± 0	3 ± 0	4 ± 0	17 ± 0	
CS	-	**♂**	-	-	-	-	-	-	-	1 ± 0	1 ± 0	1 ± 0	1 ± 0	1 ± 0	1 ± 0	-	6 ± 0	<0.01
		**♀**								-	-	1 ± 0	1 ± 0	1 ± 0	1 ± 0	-	4 ± 0	
SCS	I	**♂**	-	-	-	-	-	1 ± 0	1 ± 0	1 ± 0	1 ± 0	1 ± 0	1 ± 0	1 ± 0	1 ± 0	-	8 ± 0	<0.01
		**♀**						-	1 ± 0	1 ± 0	1 ± 0	1 ± 0	1 ± 0	1 ± 0	1 ± 0	3 ± 0	10 ± 0	
	II	**♂**	-	-	-	-	-	1 ± 0	1 ± 0	1 ± 0	1 ± 0	1 ± 0	1 ± 0	1 ± 0	1 ± 0	-	8 ± 0	<0.01
		**♀**						-	1 ± 0	1 ± 0	1 ± 0	1 ± 0	1 ± 0	1 ± 0	1 ± 0	-	7 ± 0	
BS	I	**♂**	-	-	-	-	-	-	2 ± 0	2 ± 0	2 ± 0	2 ± 0	2 ± 0	2 ± 0	2 ± 0	-	14 ± 0	<0.01
		**♀**							-	-	-	-	-	-	-	10 ± 0.42	10 ± 0.42	
	II	**♂**							-	-	-	-	-	-	-	-	-	-
		**♀**							-	-	-	-	-	-	-	1	1 ± 0	
LBS	-	**♂**	-	-	-	-	-	-	-	-	-	-	-	-	-	-	-	-
		**♀**								2 ± 0	2 ± 0	2 ± 0	2 ± 0	2 ± 0	2 ± 0	6 ± 0	18 ± 0	
SS	-	**♂**	-	-	-	-	4 ± 0.4	5 ± 0.37	8 ± 0.45	12 ± 0.56	12 ± 0.89	17 ± 0.6	17 ± 0.56	16 ± 0.09	16 ± 0.58	27 ± 0.65	135 ± 2.65	<0.01
		**♀**					5 ± 0.29	6 ± 0.65	8 ± 0.65	13 ± 0.58	15 ± 0.65	18 ± 0.58	18 ± 0.65	20 ± 0.65	24 ± 0.65	49 ± 1.11	174 ± 3.65	

Note: Values in the table are means ± standard errors (*n* = 12). Abbreviations follow Table 1. ‘-’ means without this type of sensilla. **♂**: Male; **♀**: Female.

**Table 3:** The length and width of sensilla on the antennae of male and female *Sclerodermus guani*.

Sensilla Type	Sensilla Length/μm			Basal Width (Pit Diameter)/μm			Shape	Tip	Surface	Socket
	♂	♀	*p*	♂	♀	*p*				
BB	5.45 ± 0.48	4.5 ± 0.28	0.101	0.97 ± 0.04	0.94 ± 0.06	0.635	peg (straight)	sharp	smooth	concave
TS-I (Sc, Pe)	22.35 ± 0.68	23.49 ± 0.71	0.255	1.18 ± 0.04	1.22 ± 0.05	0.593	hair (straight)	sharp	grooved	concave
TS-I (Fl)	16.51 ± 0.24	13.22 ± 0.35	<0.01	0.97 ± 0.03	0.82 ± 0.03	<0.01				
TS-II	16.07 ± 0.45	11.88 ± 0.19	<0.01	0.87 ± 0.01	0.86 ± 0.02	0.631	hair (curved)	sharp	smooth	convex
TS-III	24.34 ± 0.47	-	-	2.06 ± 0.05	-	-	hair (straight)	sharp	smooth	convex
MPS	11.25 ± 0.24	10.19 ± 0.27	<0.01	6.29 ± 0.14	5.89 ± 0.21	0.113	round	sharp/blunt	smooth	concave
CS	7.27 ± 0.19	6.64 ± 0.19	<0.05	4.41 ± 0.22	3.97 ± 0.16	0.139	round	blunt	grooved	concave
SS	1.69 ± 0.25	0.91 ± 0.16	<0.01	0.75 ± 0.08	0.54 ± 0.06	<0.01	peg (straight)	sharp	smooth	convex
BS-I	9.93 ± 0.13	8.38 ± 0.39	<0.01	1.03 ± 0.04	1.05 ± 0.05	0.743	peg (straight)	blunt	grooved	convex
BS-II	-	11.08 ± 0.23	-	-	1.70 ± 0.02	-	peg (straight)	sharp	smooth	convex
LBS	-	8.15 ± 0.27	-	-	2.45 ± 0.05	-	peg (straight)	blunt	grooved	convex
SCS-I	12.32 ± 0.18	12.77 ± 0.16	0.083	1.74 ± 0.02	1.29 ± 0.11	<0.01	rod (slightly curved)	blunt	grooved	convex
SCS-II	6.96 ± 0.19	7.05 ± 0.13	0.697	0.93 ± 0.02	0.92 ± 0.02	0.452	rod (slightly curved)	Sharp	grooved	convex

Note: Values in the table are means ± standard errors. Abbreviations follow Table 1. ‘-’means without this type of sensilla. **♂**: Male; **♀**: Female.

## Data Availability

The original contributions presented in this study are included in the article/Supplementary Materials. Further inquiries can be directed to the corresponding authors.

## Supplementary Materials

The following supporting information can be downloaded at https://www.mdpi.com/article/10.3390/insects16050547/s1, Table S1: Comparison of antennal size between female and male *Sclerodermus guani*; Table S2: Antennal sensilla of *Sclerodermus*-related species.
