# Supplementary material for: A Comparative Morphological Study of the Ultrastructure of Antennal Sensilla in Sclerodermus guani (Hymenoptera: Bethylidae)
Source: Insects. 2025 May 21;16(5):547. doi: 10.3390/insects16050547 (PMC12112000; doi:10.3390/insects16050547)
Supplement: Supplementary file 1 [file insects-16-00547-s001.zip › insects-3646404-supplementary.pdf]

Table S1. Comparison of antennal size between female and male *Sclerodermus guani*

Variable	Statistical parameter	Rd	Sc	Pe	Flagellomeres F1	F2	F3	F4	F5	F6	F7	F8	F9	F10	F11	Total
Length	*t*	-1.99	-14.08	5.05	10.91	17.93	10.06	6.91	11.16	15.16	17.68	11.53	14.87	11.03	12.73	25.55
	d.f.	18	18	18	18	18	18	18	18	18	18	18	18	18	18	18
	p	>0.05	<0.001	<0.001	<0.001	<0.001	<0.001	<0.001	<0.001	<0.001	<0.001	<0.001	<0.001	<0.001	<0.001	< 0.001
Width	*t*	2.24	-4.96	12.08	9.32	9.94	4.13	0.68	-7.57	-8.88	-8.31	-6.96	-9.02	-14.31	-13.76	-
	d.f.	18	18	18	18	18	18	18	18	18	18	18	18	18	18	-
	p	<0.05	<0.001	<0.001	<0.001	<0.001	<0.001	>0.05	<0.001	<0.001	<0.001	<0.001	<0.001	<0.001	<0.001	-

Note: The abbreviations for sensilla and antennal segments are consistent with those listed in Table 1.

Table S2. Antennal sensilla of *Sclerodermus* related species

Sensilla in this study (Abbreviations)	Citation			
	S. Sichuanensis [47]	*S. pupariae* [49]	*S. alternatusi* [50]	*S. cereicollis* [51]
BB	Basiconic sensillum I	Sensilla chaetica 2	Bohm's Bristles	-
TS-I	-	Sensilla chaetica 1	Sensilla trichodea 1/2	Trichoid sensilla
TS-II	-	-	Sensilla trichodea 3	-
TS-III	Trichoid sensillum III	Sensilla trichodea 3	Sensilla basiconica 2	Multiporous sensilla chaetica
MPS	Placodeum sensillum	Sensilla placodea	Sensilla placodea	Sensilla placodea
CS	Campaniform sensillum	Sensilla coeloconica	Sensilla coeloconica	Coeloconic pegs
SS	Squanmiformic sensillum	Sensilla squamiforma	Sensilla squamiforma	-
BS-I	-	Sensilla trichodea 2	-	Uniporous grooved sensilla chaetica
BS-II	-	Sensilla trichodea 1	-	-
LBS	Long basiconic sensillum	Sensilla basiconica	Sensilla basiconica 1	Long sensilla basiconica
SCS-I	Styloconic sensillum II	Sensilla styloconica 1	Sensilla styloconica 1	Grooved sensilla ampullacea
SCS-II	Styloconic sensillum I	Sensilla styloconica 2	Sensilla styloconica 2	Grooved peg

Note: The abbreviations for sensilla follow Table 1. "-" Displays no similar sensilla.
